# Supplementary figures and images for: Air Pollution Increases the Incidence of Upper Respiratory Tract Symptoms among Polish Children
Source: J Clin Med. 2021 May 16;10(10):2150. doi: 10.3390/jcm10102150 (PMC8156299; doi:10.3390/jcm10102150)

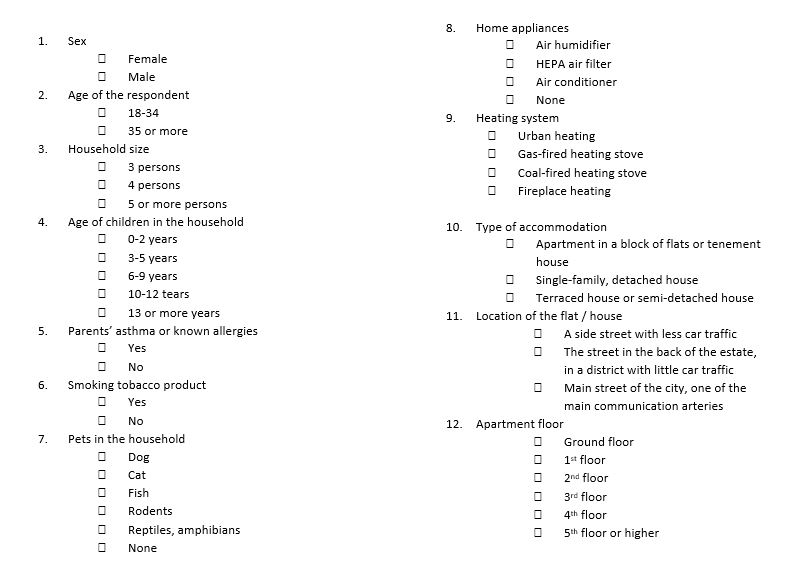

Supplement: Supplementary file 1 [file jcm-10-02150-s001.zip › fig 2.JPG]

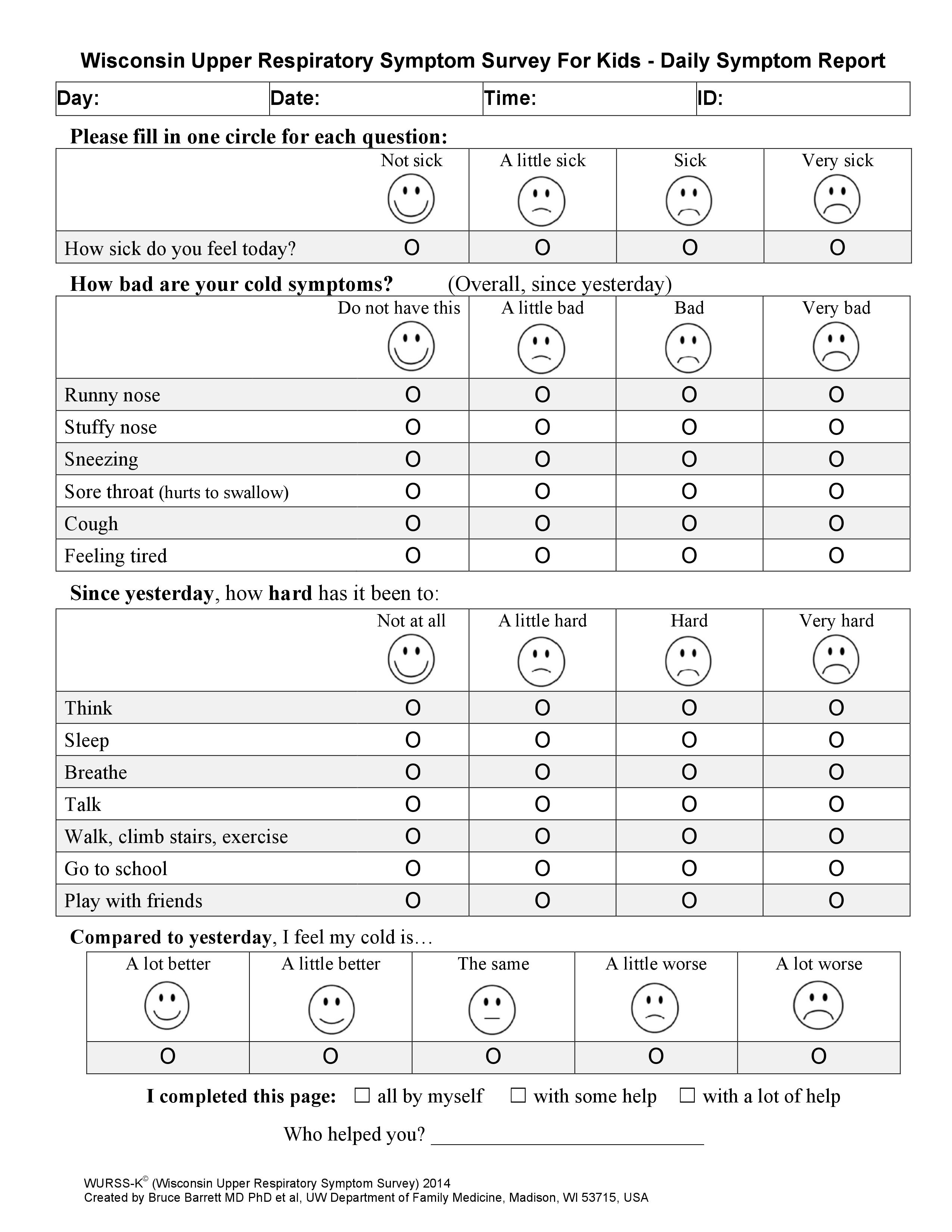

Supplement: Supplementary file 1 [file jcm-10-02150-s001.zip › fig.3 WURSS-k eng.jpg]

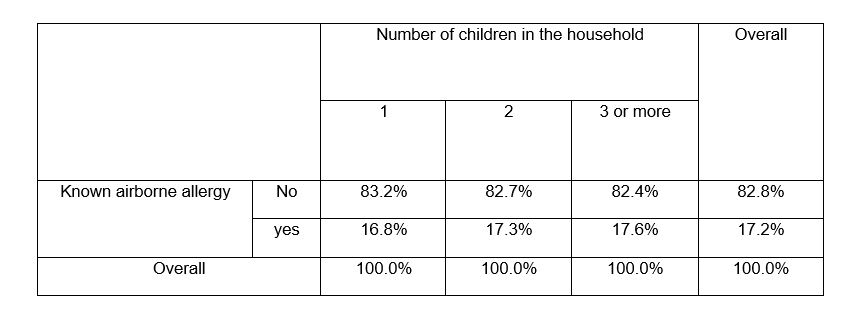

Supplement: Supplementary file 1 [file jcm-10-02150-s001.zip › supplementary material 3.JPG]
